# The circadian rhythm and core gene Period2 regulate the chemotherapy effect and multidrug resistance of ovarian cancer through the PI3K signaling pathway

**DOI:** 10.1042/BSR20202683

**Published:** 2020-11-02

**Authors:** Zhaoxia Wang, Honghong Wang, Hongrui Guo, Fengyan Li, Weiwei Wu, Sanyuan Zhang, Tong Wang

**Affiliations:** 1 Department of Obstetrics and Gynecology, First Hospital of Shanxi Medical University, Taiyuan, Shanxi, China; 2 Department of Obstetrics and Gynecology, Women Health Center of Shanxi, Taiyuan, Shanxi, China; 3 Department of Gynecology and Obstetrics, Yuncheng Central Hospital, Yuncheng, Shanxi, China; 4 School of Public Health, Shanxi Medical University, Taiyuan, Shanxi, China

**Keywords:** circadian rhythm, multidrug resistance, Ovarian cancer, Period2 gene

## Abstract

**Background**: Ovarian cancer is the most lethal cancer in the female reproductive system. It has been shown that ‘time chemotherapy’ of ovarian cancer has an important impact on the chemotherapy effect and prognosis of patients, but the specific mechanism is not known. **Methods**: We designed a case–control study in strict accordance with epidemiological principles. We collected resection samples of ovarian cancer patients who worked night-shifts and those who did not, and analyzed the differences in protein expression. Through construction of a normal/circadian-rhythm disorder model of ovarian cancer in nude mice, we explored the molecular mechanism of a ‘biological clock’ rhythm on treatment of ovarian cancer. **Results**: Expression of interleukin (IL)-6, programmed cell death receptor-1 (PD-1) and programmed death ligand 1 (PD-L1) increased, and expression of tumor necrosis factor (TNF)-α, Period 1 (*Per1*) and Period 2 (*Per2*) decreased in the night-shift group. Methylation of CpG islands in the promoter of Per2 could result in its decreased expression in SKOV3/DDP (Cisplatin) cells. Dysrhythmia of the circadian clock: (i) had a negative effect on the chemotherapy effect against ovarian cancer; (ii) affected expression of immune factors and the phosphoinositide 3-kinase/protein kinase B (PI3K/Akt) signaling pathway. **Conclusion:** The Per2 gene can affect the drug resistance of ovarian cancer by inhibiting the PI3K/Akt signaling pathway and then acting on its downstream drug-resistance factors, thereby providing a new target for ovarian cancer treatment.

## Introduction

With the hectic pace of modern life, increasing numbers of people (especially professional women) are ignoring their biological rhythms consciously to meet the demands of work. The ‘biological clock’ regulates the circadian rhythm of the human body and contributes to a series of physiological and pathological phenomena. Destruction of the circadian rhythm has been found to be closely related to the occurrence of several tumor types. For women, disorders of the biological clock caused by shift-work and other pressures can cause menstrual disorders, breast cancer and thyroid cancer [1].

Ovarian tumors are closely related to hormone secretion. Secretion of female hormones is closely related to the circadian rhythm, work and rest. Previously, we showed that the core gene of the circadian rhythm is closely related to the development of benign and malignant tumors of the ovary [2]. Overexpression of the Period 2 (Per2) gene in a tumor-transplant model in animals has been shown to improve the tumor-inhibition rate, as well as inhibit the growth and metastasis of tumor cells. Through epidemiological investigation and genomic analyses, we found that the severity of ovarian cancer is related to carrying out night-shifts and disorders of biological rhythms. Genes associated with the circadian rhythm (e.g., Period 1/2 (*Per1, Per2*)), inflammatory factors and tumor immune factors may be related to the occurrence and development of ovarian cancer.

In terms of basic research, clinicians in our research team focus on whether: (i) disorders in the biological clock caused by shift-work affect the occurrence of ovarian cancer; (ii) the treatment effect and clinical prognosis of shift-work patients with ovarian cancer will be different from that of healthy patients; (iii) basic research can provide a basis for protection for women who carry out shift-work. Therefore, we designed a case–control study strictly in accordance with epidemiological principles. We collected resection samples from ovarian cancer patients who worked night-shifts and group of ovarian cancer patients who did not work night-shifts to analyze differences in protein expression. We also used a questionnaire on night-shifts and demographic characteristics. Through the construction of a normal/circadian-rhythm disorder model of ovarian cancer in nude mice, we explored the effect of the biological clock on the treatment of ovarian cancer.

## Materials and methods

### Collection of human surgical specimens

We collected the resection samples of patients diagnosed with ovarian cancer in Shanxi Medical University (Shanxi, China) from January 2013 to December 2018 for the extraction and measurement of proteins. The study protocol was approved by the ethics review committee of Shanxi Medical University (2017LL015), and all participants provided written informed consent before approval was obtained. We provided a questionnaire on night-shifts and demographic characteristics. According to the night-shift information obtained from the questionnaire, patients with ovarian cancer were divided into two groups: ‘night-shift’ and ‘normal’. The definition of a night-shift was a working time between 11:00 p.m. and 7:00 a.m. for ≥3 times every month for ≥3 years [3,4]. We measured protein expression. We collected three resection samples from each group. Then, we undertook background matching based on the tumor stage, family income, living area, educational attainment and birth.

### Cells

The human epithelial ovarian cancer cell line SKOV3 was purchased from American Tissue Type Collection (Manassas, VA, U.S.A.) and cultured in RPMI 1640 medium (HyClone, Logan, UT, U.S.A.) and Dulbecco’s modified Eagle’s medium (HyClone). Then, 10% fetal bovine serum (FBS), 1% penicillin and 1% streptomycin were added followed by culture in a humidified atmosphere of 5% CO_2_. SKOV3/DDP cells (cisplatin-resistant) were purchased from Shanghai Yaji Biologicals (Shanghai, China) and cultured in RPMI 1640 medium supplemented with 10% FBS, 1% penicillin, 1% streptomycin and cisplatin (1 μg/ml).

### Real-time reverse transcription-quantitative polymerase chain reaction

Total RNA was isolated using TriPure™ (Roche, Basel, Switzerland) and reverse-transcribed to synthesize cDNA using M-MLV reverse transcriptase (ELK Biotechnology, Wuhan, China). Amplification was carried out using EnTurbo™ SYBR Green PCR SuperMix on a StepOne™ Real-Time PCR system (Life Technologies, Carlsbad, CA, U.S.A.). The primer sequences are shown in Table 1. Actin was used as an internal control. Relative quantification of gene expression was conducted using the 2^−ΔΔ*C*_t_^ method.

**Table 1:** Bisulfite-sequencing PCR primer

BSP	hPER2-BSP-F	GGACCTCAGGCTCCTGAGCTG	127 bp
Primer	hPER2-BSP-R	TATACCCTTAACCCTATCCCAAATAA	

ABbreviation: BSP, bisulfite-sequencing PCR.

### Animals

Forty female BALB/C nude mice (5 weeks; 14.62 ± 1.98 g) were purchased from Beijing Huafeng Biotechnology (Beijing, China) and maintained under specific pathogen-free conditions. Protocols for animal management/experiments were approved by the Ethics Committee of Shanxi Medical University. Animal experiments were conducted according to the pre-examination process stated by Shanxi Medical University, and the animal experiment was carried out in the Experimental Animal Center of Shanxi Medical University. After 1 week of acclimatization in a specific pathogen-free environment, mice were divided randomly into four groups of 10. The inoculation concentration of SKOV3 cells was 1 × 10^7^ cells/0.2 ml in serum-free RPMI 1640. Tumor formation was monitored on alternate days. Seven days later, the transplanted tumor appeared subcutaneously in nude mice. Fourteen days later, if the transplanted tumor grew to a diameter of 4−5 mm, modeling was judged to be successful. Cisplatin (3 mg/kg) was injected twice a week and via the intraperitoneal route 2 weeks after inoculation. When the tumor became visible, the length and width of the tumor was measured every week with a digital caliper, and then the tumor volume calculated using the formula: volume = (length × width)^2^/2. At the end of the experiment, mice were anesthetized by inhalation of ether and killed by cervical dislocation. Mice were killed 3 weeks after cisplatin treatment. The tumor was removed immediately and stored at −80°C until use.

### Animal grouping

The light/dark, normal light and dark environment (LD) group denoted mice exposed to a 12-h light−dark cycle. The light/light, continuous light (LL) group denoted mice exposed to light continuously for 24 h.

The ‘OC/LD’ group referred to SKOV3 cells injected subcutaneously and exposed to a normal circadian rhythm. The ‘OC/LL’ group denoted to SKOV3 cells injected subcutaneously and exposed to a circadian-rhythm disorder. The ‘R-OC/LD’ group referred to SKOV3/DDP cells injected subcutaneously and exposed to a normal circadian rhythm. The ‘R-OC/LL’ group denoted SKOV3/DDP cells injected subcutaneously and exposed to a circadian-rhythm disorder.

### Measurement of interleukin-6 and tumor necrosis factor-α contents in mouse serum by enzyme-linked immunosorbent assay

According to the specifications of the enzyme-linked immunosorbent assay (ELISA) kit, make sure that each standard substance and sample to be tested had three multiple holes. Then, the absorbance of each hole was measured at 450 nm.

### Measurement of protein expression by Western blotting

Tumor tissue was dissolved or homogenized with ice-cold dissolving buffer. Centrifugation (16000×***g*** for 10 min at room temperature) followed by heating at 95°C for 5 min in loading buffer was undertaken to collect cell lysates. Samples were separated by sodium dodecyl sulfate/polyacrylamide gel electrophoresis using 10% gels and transferred to polyvinylidene fluoride (PVDF) membranes. Then, they were sealed with 5% skimmed milk for 1 h in Tris buffer salt containing 0.1% Tween-20. Next, PVDF membranes were incubated with primary antibodies (all of which were from Abcam in Cambridge, U.K.): Per2 (1:2000 dilution), Per1 (1:500), interleukin (IL)-6 (1:1000), tumor necrosis factor (TNF)-α (1:1000), programmed cell death receptor (PD)1 (1:50), programmed cell death ligand (PD-L)1 (1:100) or β-actin (1:10000). PVDF membranes were washed thrice with phosphate-buffered saline (PBS) overnight at 4°C. Then, secondary antibodies combined with horseradish peroxidase were allowed to incubate with PVDF membranes for 1 h at room temperature. After processing using PBS, chemiluminescence signals were detected on X-ray film and quantified by ImageJ (National Institutes of Health, Bethesda, MD, U.S.A.).

### Statistical analyses

Data are the mean ± standard deviation. Prism 5 (GraphPad, San Diego, CA, U.S.A.) was employed for statistical analyses. SPSS 19.0 was used to evaluate differences between two groups. Statistical significance was assessed by the paired *t* test. *P*<0.05 was considered significant.

## Results

### Expression of genes associated with the circadian rhythm and immune factors was different between the two groups

**Figure 1:**
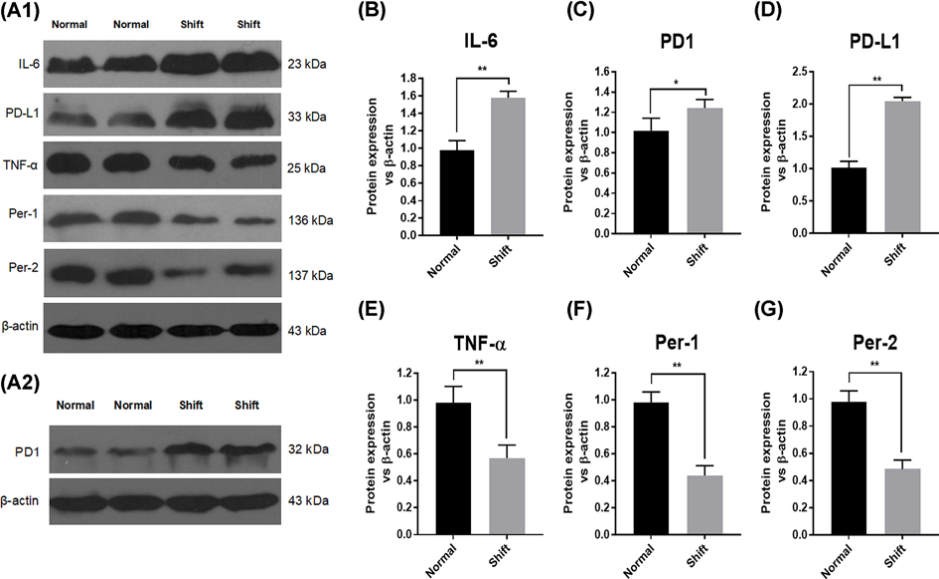
Expression of genes associated with the circadian clock and immune factors Western blotting was used to detect the rhythms and immune-related proteins of tumor samples between the normal group and night-shift group. (**A**–**G**) refers to the rhythm and immune-related protein-expression bands and respective histogram of the two groups. (A) Protein-expression bands of the two groups. Histogram of (B) IL-6, **P<0.01 vs Normal. (C) PD1, *P<0.05 vs Normal. (D) PD-L1, **P<0.01 vs Normal. (E) TNF-α, **P<0.01 vs Normal. (F) Per-1 and (G) Per2, **P<0.01 vs Normal. Expression of the tumor-promoting inflammatory factor IL-6, immunosuppressive factor PD1 and PD-L1 increased, whereas expression of the immune factor TNF-α and rhythm-related factors Per1 and Per2 decreased in the night-shift group, *P*<0.001.

Western blotting was employed to measure expression of immune system-related proteins in tumor samples between the normal group and circadian rhythm-disorder group. Expression of the tumor-promoting inflammatory factor IL-6, immunosuppressive factor PD1, and PD-L1 increased, whereas expression of the immune factor TNF-α and body rhythm-related factors Per1 and Per2 decreased in the night-shift group, and the difference was statistically significant (Figure 1A–G). This result demonstrated that night-shifts could cause disorder of expression of circadian-related regulatory factors and immune regulatory factors in the development of ovarian cancer.

### Measurement of DNA methylation of the Per2 promoter by bisulfite sequencing PCR)

**Figure 2:**
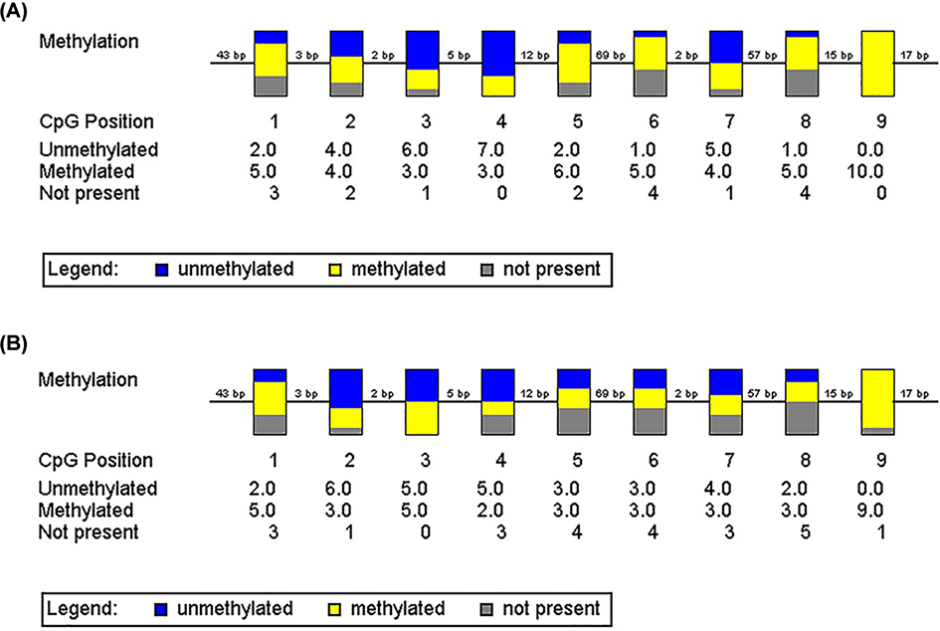
Measurement of methylation of the promoter of *Per2* gene by bisulfite-sequencing PCR To explore the possible mechanism of the loss of Per2 in SKOV3/ DDP cells, we measured the DNA methylation of CpG islands in the PER2 promoter. Methylation of the PER2 promoter at nine CpG sites in SKOV3/DDP cells (**B**) was significantly higher than that in SKOV3 cells (**A**).

To explore the possible mechanism of Per2 loss in SKOV3/DDP cells, we measured the DNA methylation of CpG islands in the Per2 promoter. Methylation of the Per2 promoter at nine CpG sites in SKOV3/DDP cells (Figure 2B) was significantly higher than that in SKOV3 cells (Figure 2A). Hence, methylation of CpG promoters may lead to a decrease in Per2 expression in SKOV3/DDP cells.

### A disorder of the biological-clock rhythm has an obvious negative effect on chemotherapy in an animal model of ovarian cancer

**Figure 3:**
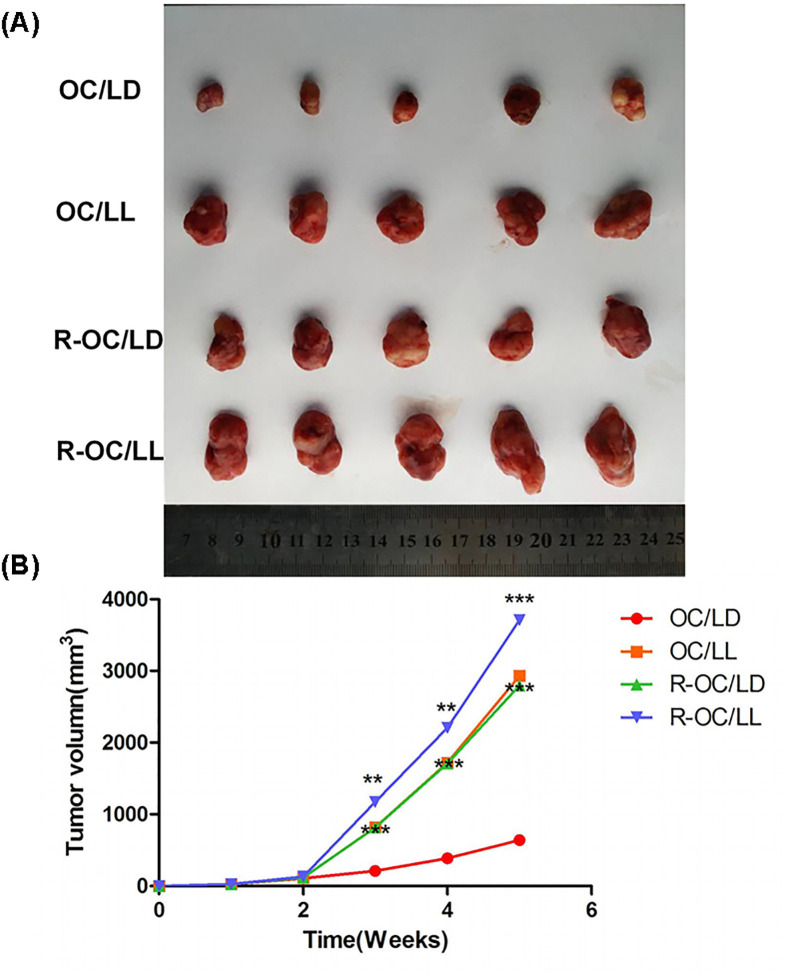
A disorder in the biological-clock rhythm has an obvious negative effect on chemotherapy in an animal model of ovarian cancer (**A**) Refers to the tumor volume of each group, while (**B**) refers to the growth curve and trend in each group. The average body weight (g) of nude mice before treatment was 16.25 ± 2.24 in the OC/LD group, 16.48 ± 2.27 in the OC/LL group, 15.97 ± 2.18 in the R-OC/LD group and 16.31 ± 2.25 in the R-OC/ LL group, respectively. There was no significant difference in body weight (*P*>0.05). Compared with the R-OC/LL group, the tumor-inhibition rate of the OC/LD group was 45.9%. Even in the R-OC/LD group, the tumor-inhibition rate was 21.9%, and the difference was statistically significant (***P<0.001). Compared with the OC/LD group, the difference in the other three groups was statistically significant (**P<0.01).

After subcutaneous injection of SKOV3 cells into nude mice, subcutaneous tumor cells were found in each group of mice 7–14 days later. When the diameter of the transplanted tumor reached 5–8 mm, the average body weight (in g) of nude mice was 16.25 ± 2.24 g in the OC/LD group, 16.48 ± 2.27 g in the OC/LL group, 15.97 ± 2.18 g in the R-OC/LD group and 16.31 ± 2.25 g in the R-OC/LL group, respectively, and these differences were not significant (*P*>0.05). The average weight and tumor-inhibition rate of each group after chemotherapy intervention are shown in Table 2. Compared with the R-OC/LL group, the tumor-inhibition rate of the OC/LD group was 45.9%. Even in the R-OC/LD group, the tumor-inhibition rate was 21.9%, and the difference was statistically significant (*P*<0.001). The change of tumor volume in each group is shown in Figure 3A,B. Compared with the OC/LD group, the difference in the other three groups was statistically significant (*P*<0.05). In the OC/LL group, the tumor volume increased rapidly, and was significantly higher than that in the OC/LD group.

**Table 2:** Average weight of transplanted tumor and tumor inhibition rate

Groups	Average weight (g)	Tumor inhibition rate (%)
OC/LD	2.922 ± 0.82	45.9*
OC/LL	4.103 ± 1.14	23.9*
R-OC/LD	4.217 ± 1.18	21.9*
R-OC/LL	5.398 ± 1.77	0

*P<0.001. Compared with R-OC/LL group, the tumor inhibition rate of other three groups was statistically significant.

### A circadian-rhythm disorder obviously affects expression of tumor-related immune factors in a mouse model

**Figure 4:**
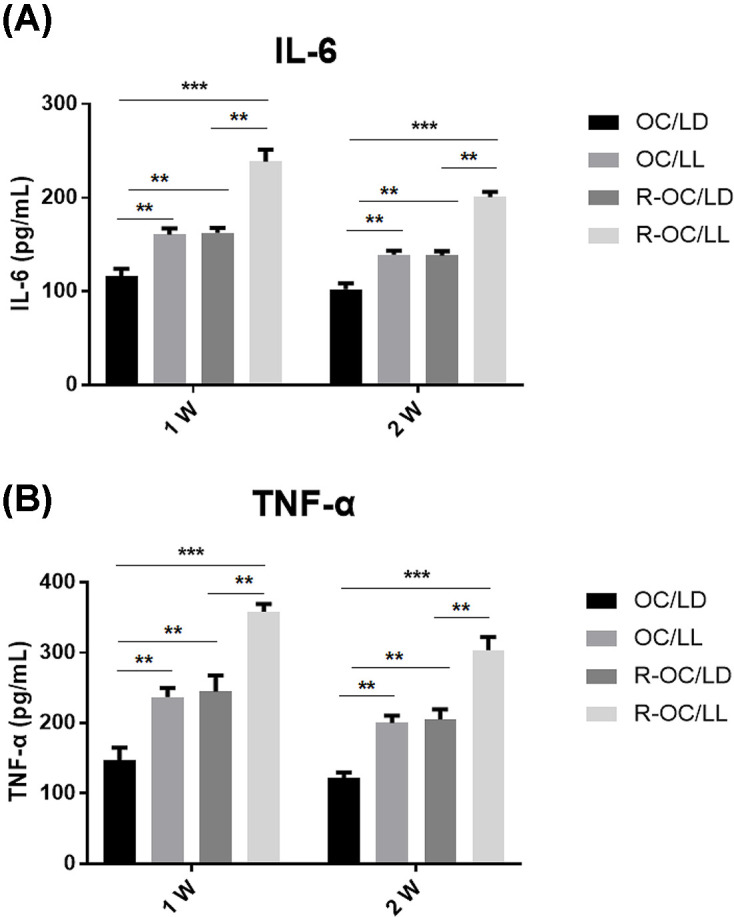
The contents of IL-6 and TNF-α in the serum of mice were detected by ELISA (**A**) Shows the change in trend of IL-6 content, and (**B**) refers to the change in trend of TNF-α content. The contents of IL-6 and TNF-α in SKOV3/DDP cells were higher than those in SKOV3 cells. The contents of IL-6 and TNF-α in the serum of mice with a circadian-rhythm disorder were higher than those of mice with a normal circadian rhythm. The F value for IL-6 was 129.63 (**P<0.01, OC/LL or R-OC/LD vs OC/LD, R-OC/LL vs R-OC/LD; ***P<0.001, R-OC/LL vs OC/LD) and the F value for TNF-α was 197.179 (**P<0.01, OC/LL or R-OC/LD vs OC/LD, R-OC/LL vs R-OC/LD; *** P<0.001, R-OC/LL vs OC/LD. Two weeks after DDP administration, the serum levels of IL-6 and TNF-α were lower than those 1 week after DDP administration.

The contents of IL-6 and TNF-α in the serum of mice were measured by ELISA. The contents of IL-6 and TNF-α in SKOV3/DDP cells were higher than those in SKOV3 cells. The contents of IL-6 and TNF-α in the serum of mice with a circadian-rhythm disorder were higher than those of mice with normal circadian rhythm. The F value for IL-6 was 129.63 (*P*<0.001) and for TNF-α was 197.179 (*P*<0.001). Two weeks after DDP administration, the serum levels of IL-6 and TNF-α were lower than that at 1 week after DDP administration (Tables 3 and 4; Figure 4A,B).

**Table 3:** IL-6 expression (pg/ml) in different groups

Groups	1 week				2 weeks			
	OC/LD	OC/LL	R-OC/LD	R-OC/LL	OC/LD	OC/LL	R-OC/LD	R-OC/LL
Mean	116.384	160.605	162.497	238.680	102.261	139.092	138.347	200.610
SD	7.847	6.534	5.311	12.703	6.089	4.572	4.512	5.617

**Table 4:** TNF-α expression (pg/ml) in different groups

Groups	1 week				2 weeks			
	OC/LD	OC/LL	R-OC/LD	R-OC/LL	OC/LD	OC/LL	R-OC/LD	R-OC/LL
Mean	147.339	236.899	244.614	358.022	121.680	200.200	204.979	303.575
SD	17.473	12.784	22.999	11.301	8.052	10.329	14.370	18.751

### Measurement of protein expression in tumor tissue by western blotting

**Figure 5:**
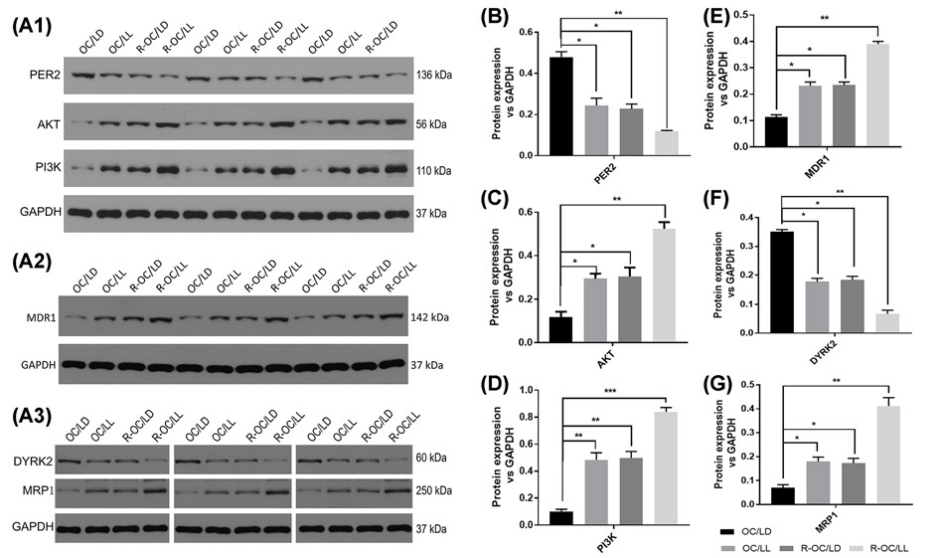
Western blotting of protein expression in tumor tissue (**A–G**) Refers to the expression bands and respective histograms of rhythm- and drug-resistance related proteins in tumor tissue. (A) Shows the protein-expression bands in the four groups. Histogram of (B) PER2, (C) Akt, (D) PI3K, (E) MDR1, (F) DYRK2 and (G) MPR1. Western blotting showed that expression of Akt, PI3K, MDR1 and MRP1 in tumor tissue injected with SKOV3/DDP cells was higher than that in tumor tissue injected with SKOV3 cells (DYRK2 expression was lower). Expression of Akt, PI3K and MDR1 in tumor tissue exposed to a circadian-rhythm disorder was higher than that in tumor tissue exposed to a normal circadian rhythm (DYRK2 expression was lower) and the difference was statistically significant (*P*<0.01). Expression of Per2 in SKOV3/DDP cells was lower than that in SKOV3 cells, whereas expression of Per2 in dysrhythmic tumors was lower than that in normal tumors, and all differences were statistically significant (*P*<0.01).

Western blotting results (four groups, three repetitions in each group) are shown in Figure 5. Expression of phosphoinositide 3-kinase (PI3K), protein kinase B (Akt), multidrug resistance gene 1 (*MDR1*) and multidrug resistance-related protein 1 (MRP1) in tumor tissue injected with SKOV3/DDP cells was higher than that in tumor tissue injected with SKOV3 cells (expression of dual-specificity tyrosine phosphorylation-regulated kinase 2 (DYRK2) was lower). Expression of Akt, PI3K and MDR1 in tumor tissue exposed to a circadian-rhythm disorder was higher than that in tumor tissue exposed to a normal circadian rhythm (DYRK2 expression was lower), and the difference was statistically significant (*P*<0.01). Expression of Per2 in SKOV3/DDP cells was lower than that in SKOV3 cells, whereas expression of Per2 in dysrhythmic tumors was lower than that in normal tumors, and all differences were statistically significant (*P*<0.01).

## Discussion

The ovary is an important organ affected by hormones and which produces hormones in women. The occurrence of ovarian cancer is closely related to hormone secretion. Female hormones are regulated by the circadian rhythm and the monthly rhythm. Disruption of the circadian rhythm is associated with an increased risk of tumor development. Epidemiological studies have shown that shift-work schedules are associated with a high risk of fatal ovarian cancer in women [5]. The International Agency for Research on Cancer has stated that abnormal changes in the genes associated with the biological clock have the same carcinogenic effect as those elicited by the human papillomavirus, diesel engines and inorganic lead compounds in exhaust gases. Genes associated with the circadian rhythm show high expression in the ovary (which regulate ovulation), and a disorder of the circadian rhythm is related to several risk factors of ovarian cancer. However, detection of variation in the circadian rhythm in germ cells as a predictor of the risk of ovarian cancer and invasion of ovarian cancer cells has not been studied.

The *Per2* gene is an important gene associated with the biological clock. Several studies have shown that abnormal expression of the *Per2* gene has an important role in the occurrence and development of cancer [6,7]. Per2 can regulate expression of several downstream genes, such as cyclin a, cyclin B1, cyclin D1, cyclin E, p53 and c-myc [8]. Therefore, it has been postulated that abnormal expression of Per2 can lead to the malignant transformation of cells by changing the response of the processes and monitoring points of the cell cycle to DNA damage.

Western blotting of resection specimens revealed that expression of the *Per2* gene in the night-shift group was down-regulated significantly. As an important core gene of the circadian clock, expression of Per2 is absent or decreased from tumor cells. Per2 expression is regulated by the methylation level in its promoter region [9]. Our results showed that the methylation level of the promoter region of the *Per2* gene in SKOV3/DDP cells was significantly higher than that in SKOV3 cells. Hence, methylation of CpG islands may lead to a decrease in Per2 expression in SKOV3/DDP cells. Expression of IL-6, PD1 and PD-L1 was up-regulated in the night-shift group. The oncogene IL-6 promotes the development of cancer through inflammatory signals. IL-6 is also a risk factor of reproductive system tumors. PD-L1/PD1 is the most common tumor-immunosuppressive signal, which contributes to the development of tumors in the female reproductive system and is used to predict the prognosis [10]. In addition, expression of TNF-a was lower in night-shift patients, which was consistent with the down-regulation of recombinant human TNF receptor superfamily member 6B (TNFRSF6B) expression in the night-shift group. We concluded that the TNF-α signal in the night-shift group was weakened/blocked. We believe that TNFRSF6B is a tumor suppressor in ovarian cancer. In a subsequent cohort study, we will compare the survival rate of two groups of ovarian cancer patients (high TNFRSF6B expression and low TNFRSF6B expression), and the exact mechanism by which the TNF-α/TNFRSF6B signaling pathway inhibits ovarian cancer. Making a reliable judgment of the prognosis and survival analysis will aid clinical practice.

The circadian rhythm is not only involved in the occurrence of ovarian cancer, but is also related to chemotherapy resistance. Inhibition of expression of the genes involved in the circadian rhythm can reverse the cisplatin resistance of SKOV3/DDP cells by influencing the protein expression of drug-resistant genes [11]. In addition, the peak of DNA synthesis is very different between malignant cells and benign cells, and there are obvious differences related to the circadian rhythm between tumor-cell proliferation and non-tumor-cell proliferation [12]. Those observations suggest that the circadian rhythm is involved in the development of drug resistance in ovarian cancer. According to the difference in the biological-clock mechanism between normal cells and cancer cells, chemotherapy combined with the circadian rhythm could reduce chemotherapy resistance, and improve the curative effect or aid the prognosis [13].

We found that a model which is sensitive to chemotherapy drugs and which exists in a stable circadian rhythm had the best therapeutic effect. A model sensitive to chemotherapy drugs but with a circadian-rhythm disorder had a much lower therapeutic effect, close to that of a model of drug resistance with a stable circadian rhythm. Therefore, the circadian rhythm has an important role in the treatment of even non-drug-resistant tumors. We also measured expression of the related proteins that may affect the development of tumor cells and the mechanism of drug resistance. Expression of Akt, PI3K, MDR1 and MRP1 in the tumor tissues of the drug-resistant group was higher than that in the tumor tissues of the cisplatin-sensitive group (DYRK2 expression was lower), which are the proteins related to drug resistance.

Expression of Akt, PI3K and MDR1 in tumor tissue exposed to a circadian-rhythm disorder was higher (DYRK2 expression was lower) than that of tumor tissue exposed to a normal circadian rhythm. Hence, even in a cisplatin-sensitive group, a circadian-rhythm disorder will lead to drug resistance of the tumor and a decrease in the therapeutic effect. Expression of Per2 in drug-resistant tumor tissue was lower than that in cisplatin-sensitive tumor tissue, and that in dysrhythmic tumor tissue was lower than that in tumor tissue exposed to a normal circadian rhythm. Our results show that the genes associated with a circadian rhythm have an important role in tumor chemotherapy, which may affect drug resistance through the PI3K/Akt signaling pathway and its important downstream factors (e.g., dyrk2, MDR1).

Per2 has been reported to have antitumor and chemosensitivity-enhancing effects [14]. It can regulate the time-dependent expression of cell cycle-related genes, inhibit angiogenesis and have an antitumor role [15]. Mutations or deletions in the *Per2* gene during tumorigenesis, and Per2 overexpression can regulate expression of the genes associated with the cell cycle and apoptosis, which have a role in cancer suppression and cisplatin treatment [16,17]. Per2 can significantly down-regulate expression of PI3K and Akt, and the PI3K signaling pathway has been demonstrated to be involved in the regulation of chemotherapy resistance in several studies [18,19]. It has been found that hepatocyte growth factor (HGF) in the tumor microenvironment can lead to chemotherapy resistance in ovarian cancer by up-regulating expression of the PI3K/Akt signaling pathway [20]. Also, the Tribbles pseudokinase 2 gene can affect the chemotherapy resistance of tumors by activating Akt of the PI3K signaling pathway [21]. The upstream and downstream parts of the PI3K pathway involve multiple genes or proteins involved in the drug-resistance mechanism of ovarian cancer. The *Per2* gene can significantly regulate expression of the PI3K/Akt signaling pathway and act on its downstream parts and drug-resistance factors. In this way, it has an important role in the invasion, metastasis and multidrug resistance of tumor cells [22,23]

Here, we found that the effect of the circadian rhythm on the chemotherapy of ovarian cancer was consistent with the concept of ‘time chemotherapy’ proposed by international organizations focused on cancer research [24]. The physiological peak time of DNA synthesis in ovarian cancer is very different between malignant cells and benign cells. For example, if a drug is given in a specific period of time to match the high vulnerability of tumor cells and the low toxicity to normal tissues, the effect will be better [12]. Time chemotherapy refers to choosing the correct time to match the individual biological clock, improving the curative effect, as well as reducing drug resistance and toxicity to prolong patient survival. Even patients with advanced gynecological and urogenital cancer can benefit from the optimal day-and-night cycle of chemotherapy, which can reduce chemotherapy resistance, and improve treatment efficacy or the prognosis [25]. Those studies indicate that the circadian rhythm has an important role in cancer treatment.

## Conclusions

A disorder of the circadian rhythm caused by shift-work is closely related to the formation and development of ovarian tumors [26]. A gene associated with the biological clock, *Per2*, can inhibit the growth and metastasis of ovarian cancer cells [27]. Whether an abnormal circadian rhythm influences the chemotherapy effect and prognosis of patients with ovarian cancer has not been reported previously. The clinical effect of time chemotherapy for ovarian cancer and how genes associated with the circadian affect the drug resistance of ovarian chemotherapy merits further study.

## Data Availability

Research data can be obtained by contacting the first author or corresponding authors: Zhaoxia Wang, 13835181196@163.com or Tong Wang, tongwang@sxmu.edu.cn.

## Abbreviations

Akt: protein kinase B
DDP: cisplatin
DYRK2: dual-specificity tyrosine phosphorylation-regulated kinase 2
ELISA: enzyme-linked immunosorbent assay
FBS: fetal bovine serum
IL: interleukin
LD: light/dark, normal light and dark environment
LL: light/light, continuous light
*MDR1*: multidrug resistance gene 1
M-MLV: moloney murine leukemia virus
MRP1: multidrug resistance-related protein 1
OC: ovarian cancer
PD: programmed cell death receptor
PD-L: programmed cell death ligand
*Per1/2*: period 1/2
SKOV3: Aa cell line of ovarian adenocarcinoma
TNF: tumor necrosis factor
TNFRSF6B: TNF receptor superfamily member 6B
